# Hoof Diseases

**Published:** 1902-01

**Authors:** W. E. A. Wyman


### DEPARTMENT OF SURGERY.

By W. E. A. Wyman, M.D.V., V.S.,
MILWAUKEE, WIS.

### Hoof Diseases.

(Continued from page 426, July, 1901.)

Bruised, incised, punctured, etc., wounds of the pododerm, as the result of forging, nail-pricks, cracked hoof-calks, are the most common point of entrance of the pus-producing germs. Probably the most frequent cause is the senseless deep paring of so-called corns. Since in both serous and hemorrhagic pododermatitis a ready medium for the development of bacteria is offered, it is readily seen that just such cases presenting a bruised state of the pododerm are especially liable to be infected. Finally, as a matter of contiguity, infection of neighboring parts follows suppurative processes, as suppurative dermatitis, quittors, etc. For reasons previously discussed, the hoof of the anterior extremities—and here, again, the internal aspect—are more frequently exposed to the injuries just enumerated, and thus more frequently attacked by suppurative pododermatitis. Another reason for the accumulation of pus—at least concerning injuries of the fleshy wall, sole, and frog—lies in the fact that the wound closes early by the inhibition and consequent swelling of the horn cells by the liquid products of the inflammation.

The temperature, the moisture, and the presence of a rich blood-supply represent an ideal medium for the development of bacteria and their toxins in the hoof. They multiply rapidly and invade the surrounding parts. Their metabolistic products first create a hyperæmic state, followed by serous exudation and infiltration of white and red blood cells into the tissues ; pus-producing bacteria enter in some way or other, and a purulent exudate occurs. In superficial suppurative pododermatitis the purulent exudate permeates the surface of the sensitive laminæ, and here, in the stratum mucosum, the pus separates the cells of the rete Malpighii, thus eventually disconnecting the fleshy and horny laminæ; pressure is then brought to bear upon these parts, and anæmia follows. By the application of operative measures the pus can be liberated, as it now rests between the horny capsule and the papillary body.

When not relieved the process destroys more and more cells of the stratum mucosum; therefore in the region of the sensitive laminæ of the wall the pus eventually appears at the coronet. In the region of the fleshy sole the white line forms the point of exit, while the product of superficial suppurative pododermatitis of the fleshy frog may empty along the coronary region of the heel.

The pus of superficial suppurative pododermatitis consists of pus corpuscles, pus germs, and serous exudate; therefore it is thin and, on account of the admixture of the pigmented rete cells, gray or black. It is usually limited in quantity, at first of alkaline reaction and odorless, while the presence of putrefactive bacteria will develop a decided malodor.

Superficial suppurative pododermatitis, unlike an aseptic pododermatitis, more rarely terminates in resolution, while chronic inflammation, deep suppurative, and even gangrenous pododermatitis are possibilities. In simple resolution resorption of the products of inflammation occurs, provided no pus has as yet accumulated; in other words, resolution is only possible in case of purulent infiltration of the tissues. Accumulation of pus is to be expected. At first the pressure exerted by the pus upon the papillary body renders the same anæmic. As soon as the pus is evacuated, and the pressure thus removed from the papillary body, a congestive hyperæmia follows, which in return means an active cell proliferation in the rete Malpighii. These newly formed cells, so to speak, cap the sensitive villi, and as new cells form the first-formed cells are moved toward the wall or sole, as the case may be, reaching the sole or wall in time and destroying any effect, provided the amount of tissue destroyed by the inflammatory process was limited; otherwise, cavities of variable size, filled with a smeary black mass, are the result.

In those cases with active destruction of tissue by the purulent process, involving a greater portion of the papillary body, with retention of pus, cell proliferation, so essential to restore an equilibrium—that is, granulations—is continually wiped out, and depending on the site of the purulent process—for instance, the sensitive wall laminæ—and a tubular ulcer or fistula is the consequence.

Deep suppurative pododermatitis, as a sequel to the superficial form, is not often seen, and is most likely to follow retention of pus.

Gangrenous pododermatitis of a superficial type is also rare, and, when met with, arises usually independently as the result of traumatism. More or less suppurating leg lameness, quite often setting in suddenly, is the first symptom. The writer had many cases of superficial suppurative pododermatitis where this pododermatitis undoubtedly existed longer than the lameness, as the amount of pus found on opening the hoof was entirely out of proportion to the date of lameness. As in all suppurating leg lameness arising in the hoof, going down hill, continuous exercise, and work on hard ground increase lameness. In those cases where the pododerm about the heels is diseased the animal exhibits volar flexion of the phalanges—that is, it knuckles over—while a peculiar snatchy motion is observed when the pododerm about the toe region of the hind hoof is involved.

In the standing posture pain causes the creature to shift the hoof from one spot to another, and, as just stated, it will show volar flexion of the lower phalanges when the pododerm about the heel is diseased. The general health of the animal is seldom upset; very nervous horses at times appear somewhat depressed.

Palpation with the hand exhibits abnormal heat, while a circumscribed, very painful spot is shown by the hammer or pincers. Œdema of the subcutaneous tissues of the lower phalangeal region, depending upon a distributed circulation and that condition *sine qua non*—namely, a strongly beating phalangeal pulse—represents the *quod erat demonstrandum.* In those cases where the purulent exudate is still within the horny capsule—that is, has not yet burrowed itself into the outer world—one finds a circumscribed brown or black horny area, best seen along the white line, sole, inflection of the bars, especially in the white hoof. As soon as the paring knife permits its escape, a thin, blackish hoof pus appears. Of course, in those cases where the pododerm about the coronary region is undergoing the purulent process, simple inspection of the parts suffices to make the diagnosis. The same holds true when the pus escapes through solutions of continuity, as cracked hoof, nail-pricks, etc.

The diagnosis of superficial purulent pododermatitis therefore rests upon the above ensemble. The thinness and gray color of the hoof pus is also characteristic of the superficial type of purulent pododermatitis. The differential diagnosis takes into consideration corns and the two forms of aseptic pododermatitis, viz., the serous and the hemorrhagic one. Corns *per se* are not productive of lameness; the red spots are circumscribed. In serous pododermatitis the discolored horn is yellow, while a radiating red discoloration is peculiar to hemorrhagic pododermatitis. (See chapters on Corns, Serous and Hemorrhagic Pododermatitis.)

From an economic stand-point the chances for a recovery are important. In making the prognosis the extent, intensity, and the seat of the affection, its duration previous to the moment of examination, and the type of hoof (wide, drop sole, etc.), as well as the indirect causes (nail-pricks, cracked hoof, calking) are of special interest. To be sure, resolution is fortunately the more frequent termination, and thus a favorable diagnosis is warranted; yet the possibilities of a deep pododermatitis and necrosis, with subsequent septicæmia or pyæmia, must not be lost sight of. The presence of a chronic inflammation—that is, ulceration or a fistula—call for an unfavorable diagnosis.

